# The efficacy and safety of continuous intravenous tirofiban for acute ischemic stroke patients treated by endovascular therapy: a meta-analysis

**DOI:** 10.3389/fneur.2024.1286079

**Published:** 2024-04-03

**Authors:** Mengmeng Wang, Jing Li, Lingyu Zhang, Nana Li, Xuemei Li, Pengfei Wang

**Affiliations:** ^1^Clinical College, Shandong Second Medical University, Weifang, China; ^2^Department of Neurology, Weihai Municipal Hospital, Cheeloo College of Medicine, Shandong University, Weihai, Shandong, China; ^3^Department of Neurology, School of Clinical Medicine, Affiliated Hospital of Weifang Medical University, Weifang Medical University, Weifang, China

**Keywords:** tirofiban, endovascular therapy, stroke, continuous intravenous administration, meta-analysis

## Abstract

**Introduction:**

Tirofiban is a non-peptide selective glycoprotein IIb/IIIa receptor inhibitor with a short half-life. The research assesses the efficacy and safety of continuous intravenous tirofiban in patients with acute ischemic stroke (AIS) undergoing endovascular therapy (ET).

**Methods:**

A systematic search of Pubmed, Embase, Web of Science, and Cochrane Library databases is conducted from inception until January 26, 2024. Eligible studies are included based on predefined selection criteria. Efficacy outcomes (favorable functional outcome and excellent functional outcome) and safety outcomes (symptomatic intracranial hemorrhage [sICH], any intracranial hemorrhage [ICH], and 90-day mortality) are calculated using odds ratios (OR) and 95% confidence intervals (CI).

**Results:**

A total of 4,329 patients from 15 studies are included in the analysis. The results indicate a significant trend toward favorable functional outcomes in the tirofiban group (OR, 1.24; 95% CI, 1.09–1.42; *p* = 0.001). In terms of safety outcomes, tirofiban does not increase the risk of sICH (OR, 0.90; 95% CI, 0.71–1.13; *p* = 0.35) or any ICH (OR, 0.97; 95% CI, 0.70–1.34; *p* = 0.85), but it significantly decreases 90–day mortality (OR, 0.75; 95% CI, 0.64–0.88; *p* = 0.0006). A subgroup analysis suggests that continuous intravenous tirofiban demonstrates better efficacy (OR, 1.24; 95% CI, 1.09–1.42; *p* = 0.001) for patients with AIS undergoing rescue ET with even better results when used in combination with intra–arterial and intravenous administration (OR, 1.25; 95% CI, 1.07–1.451; *p* = 0.005).

**Conclusion:**

Continuous intravenous tirofiban is effective and safe for patients with AIS undergoing rescue ET, particularly when combined with intra-arterial tirofiban.

**Systematic review registration:**

PROSPERO, identifier CRD42023385695.

## 1 Introduction

As endovascular therapy (ET) technology advances, previous research has demonstrated that ET is a secure and efficient medical intervention for patients with acute ischemic stroke (AIS) caused by large vessel occlusion (LVO) (1). Currently, ET has become a crucial element in the standard treatment protocol due to its proven association between improved functional outcomes and reduced mortality compared to the traditional medical therapy. This improvement is achieved through the direct removal of thrombus and a significant increase in the successful reperfusion rates. However, investigations have demonstrated that reocclusion continues to occur even after recanalization in approximately 20% individuals, leading to an increased risk of a poor prognosis beyond 90 days (2). The reocclusion is often attributed to endothelial injuries and platelet aggregation during the ET procedure, which is hardly avoidable (3). In addition, the rupture of atherosclerotic plaques during ET results in the downstream migration of plaque fragments, which inflicts multiple insults on the cerebral microcirculation. Therefore, early bridging antiplatelet treatment during ET has gained significant interest.

Glycoprotein (GP) IIb/IIIa inhibitors act on the surface of platelet, playing a key role in blocking the final common pathway to platelet aggregation. Tirofiban, a non-peptide compound that explicitly inhibits the GP IIb/IIIa receptor involved in platelet aggregation, has a short half-life. This drug exhibits distinct antiplatelet effects due to its unique pharmacokinetic properties. Tirofiban is currently used in individuals with acute coronary syndromes. Qiu et al. conducted a randomized, double-blind, placebo-controlled trial, which did not provide evidence to support the use of intravenous tirofiban before ET for patients with AIS (4). Furthermore, opinions regarding tirofiban treatment in patients with AIS undergoing ET have been inconsistent (5, 6). More information is needed to address the efficacy and safety of continuous intravenous tirofiban in patients with AIS treated with ET. Depending on the clinical situation, tirofiban is administered in different ways, such as bolus intravenous administration, bolus intra-arterial administration, and continuous intravenous administration. A continuous infusion refers to the administration of the entire tirofiban dose without any interruption over a dosing period. The continuous intravenous administration encompassed three modalities, namely continuous intravenous alone, the combination of bolus intra-arterial with continuous intravenous, and the combination of bolus intravenous with continuous intravenous. Continuous intravenous administration of tirofiban effectively prevents reocclusion and protects against the microcirculation. This effectiveness is due to the ability of tirofiban to adjust the dose and maintain steady efficacy in inhibiting platelet aggregation. Therefore, continuous intravenous administration of tirofiban has been the subject of numerous research studies. This research aims to assess the efficacy and safety of continuous intravenous tirofiban in patients with AIS who undergo ET.

## 2 Materials and methods

### 2.1 Search strategy

The search protocol adheres to the Preferred Reporting Items for Systematic Reviews and Meta-Analyses (PRISMA) guidelines (7). The databases, such as Pubmed, Embase, Web of Science, and Cochrane Library, are searched for studies published from the inception of the databases until January 26, 2024. The mesh terms “Stroke” and “Tirofiban” are utilized, and their corresponding free terms are systematically employed (Supplementary material 1). The search is performed by two authors (Mengmeng Wang and Jing Li) independently via screening the titles and abstracts of articles based on predefined inclusion and exclusion criteria. This meta-analysis has been registered in PROSPERO (ID: CRD42023385695).

### 2.2 Selection criteria

This study included the following criteria for inclusion: (1) prospective or retrospective cohort study or randomized controlled trials (RCTs); (2) patients diagnosed with AIS who receive ET within 8 h for occlusions in the anterior circulation and within 24 h for occlusions in the posterior circulation following the onset of stroke symptoms; and (3) the patient division into two groups based on whether they receive intravenous tirofiban throughout the operation.

This study included the following criteria for exclusion: (1) single-arm trials; (2) animal studies, case reports, conference abstracts, or reports where data cannot be gathered from the published article; (3) studies involving only intra-arterial tirofiban throughout the operation; (4) studies from the same database; and (5) studies with fewer than 30 participants per group.

### 2.3 Outcomes

The efficacy outcomes include favorable and excellent functional outcomes at 3 months. A favorable functional outcome is defined as modified Rankin Scale (mRS) 0–2, indicating self-sufficiency and independence in daily activities at 90 days. Meanwhile, the excellent functional outcome is defined as mRS 0-1, indicating proficiency in performing usual responsibilities and tasks at 90 days. The safety outcomes include symptomatic intracranial hemorrhage (sICH), any intracranial hemorrhage (ICH), and 90-day mortality. sICH and any ICH are determined according to the ECASS III study (8) or the Heidelberg Bleeding Classification (9). The 90-day mortality is defined as an mRS score of 6 within 3 months.

### 2.4 Data extraction and quality assessment

Two researchers independently extract the data, which are then cross-checked for consistency. In the event of any disagreement, a discussion between the two researchers is held to resolve the issue. If necessary, the assistance of a third professional is sought. The following details are recorded: author (year), country, study design, sample size, sex, age, intravenous thrombolysis, onset to recanalization time (OTR), onset to puncture time (OTP), administration route of tirofiban, cause of stroke, occlusion location in the cerebral artery, efficacy outcomes, and safe outcomes. The Newcastle-Ottawa Scale (NOS) is used to assess the quality of observational studies based on three domains: selection, comparability, and result of the research (10). The time of stroke onset is defined as the last recorded instance when the patient was observed to be in a normal condition. The stroke causative mechanism is assessed based on the Trial of ORG 10172 in Acute Stroke Treatment (TOAST) classification (11). The Cochrane Risk of Bias Tool (RoB 2) is utilized to evaluate the quality of RCTs, categorizing the trials into low, medium, and high quality (12). The Newcastle-Ottawa Scale (NOS) is used to assess the quality of observational studies based on three domains, namely selection, comparability, and result of the research. A study with a NOS score of 7–9 is considered high quality (10).

### 2.5 Statistical analysis and sensitivity analysis

The statistical analysis is conducted using RevMan 5.3 software provided by the Cochrane Collaboration. Each outcome is represented by a binary variable. The odds ratios (OR) with 95% confidence intervals (CI) are generated using a fixed-effects model, and the Mantel–Haenszel technique is used to determine the effect size. Statistical significance is defined as a *p* < 0.05. Meanwhile, the heterogeneity is assessed using the weighted sum of squared differences (I^2^). I^2^ values over 75% indicate high heterogeneity, while values ranging from 50% to 75% indicate moderate heterogeneity. In light of the prevailing conditions, a random-effects model is employed. To assess the influence of individual studies on the overall findings, a sensitivity analysis is conducted by systematically excluding one study at a time. Publication bias is assessed using funnel plots.

## 3 Results

### 3.1 Search results and study characteristics

A total of 666 potentially relevant publications were retrieved by two researchers independently. After excluding duplicate entries and evaluating the titles and abstracts, a total of 58 articles were identified for further examination of the full texts. In the present investigation, the study with the largest sample size was selected from a pool of studies from the same database. Ultimately, this meta-analysis incorporates a total of 15 papers that meet the inclusion criteria of the current research work (Supplementary material 2). Among these, one is a RCT study (4), 10 are retrospective studies (13–22), and the last four are prospective studies (23–26). A total of 4,329 patients from 15 selected studies are included. The baseline characteristics of the included studies and the quality assessment of these studies are summarized in Supplementary material 3. All of the included studies are considered to be high quality (Supplementary materials 4, 5).

### 3.2 Efficacy outcomes

#### 3.2.1 Favorable functional outcome

**Figure 1:**
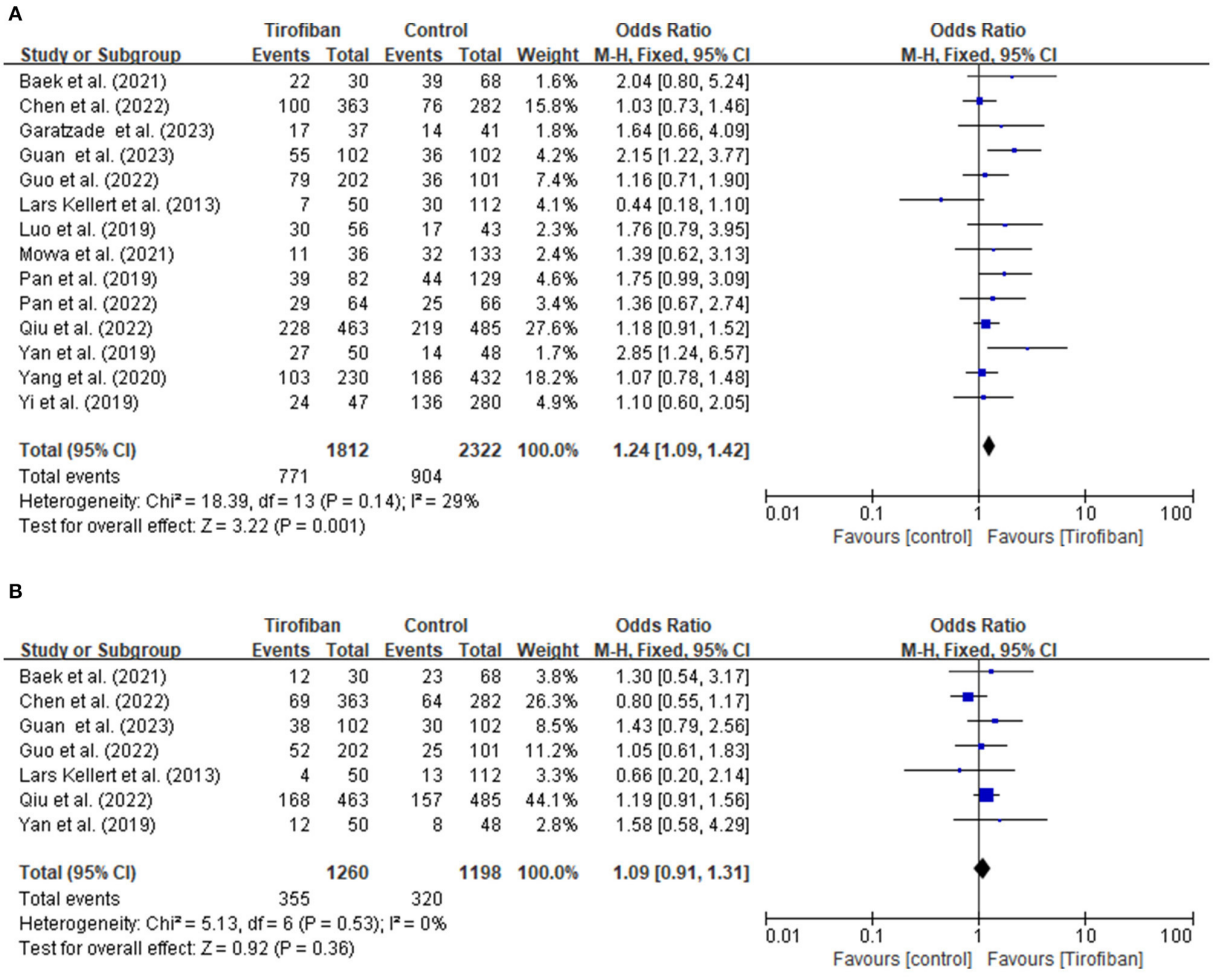
**(A, B)** Forest plot of tirofiban vs. control for efficacy outcomes meta-analyses of related studies.

Fourteen studies report a favorable functional outcome at the 3-month follow-up (Figure 1A). The incidence of a favorable functional outcome is 42.5% (771/1,812) in the tirofiban group and 38.9% (904/2322) in the non-tirofiban group. The pooled analysis demonstrates a statistically significant increase in the probability of achieving a favorable functional outcome in the tirofiban group (OR, 1.24; 95% CI, 1.09–1.42; *p* = 0.001), without any significant heterogeneity (*p* = 0.14; I^2^ = 29%).

#### 3.2.2 Excellent functional outcome

Seven research studies provide data for evaluating the excellent functional outcome at the 3-month follow-up period (Figure 1B). The incidences of achieving an excellent functional outcome in the tirofiban and non-tirofiban groups are 28.2% (355/1,260) and 26.7% (320/1,198), respectively. The pooled analysis reveals no statistically significant difference in excellent functional outcome between the patients in the tirofiban and non-tirofiban groups (OR, 1.09; 95% CI, 0.91–1.31; *p* = 0.36). No significant heterogeneity is detected among the trials included in this analysis (*p* = 0.53; I^2^ = 0%).

### 3.3 Safety outcomes

#### 3.3.1 sICH incidence

**Figure 2:**
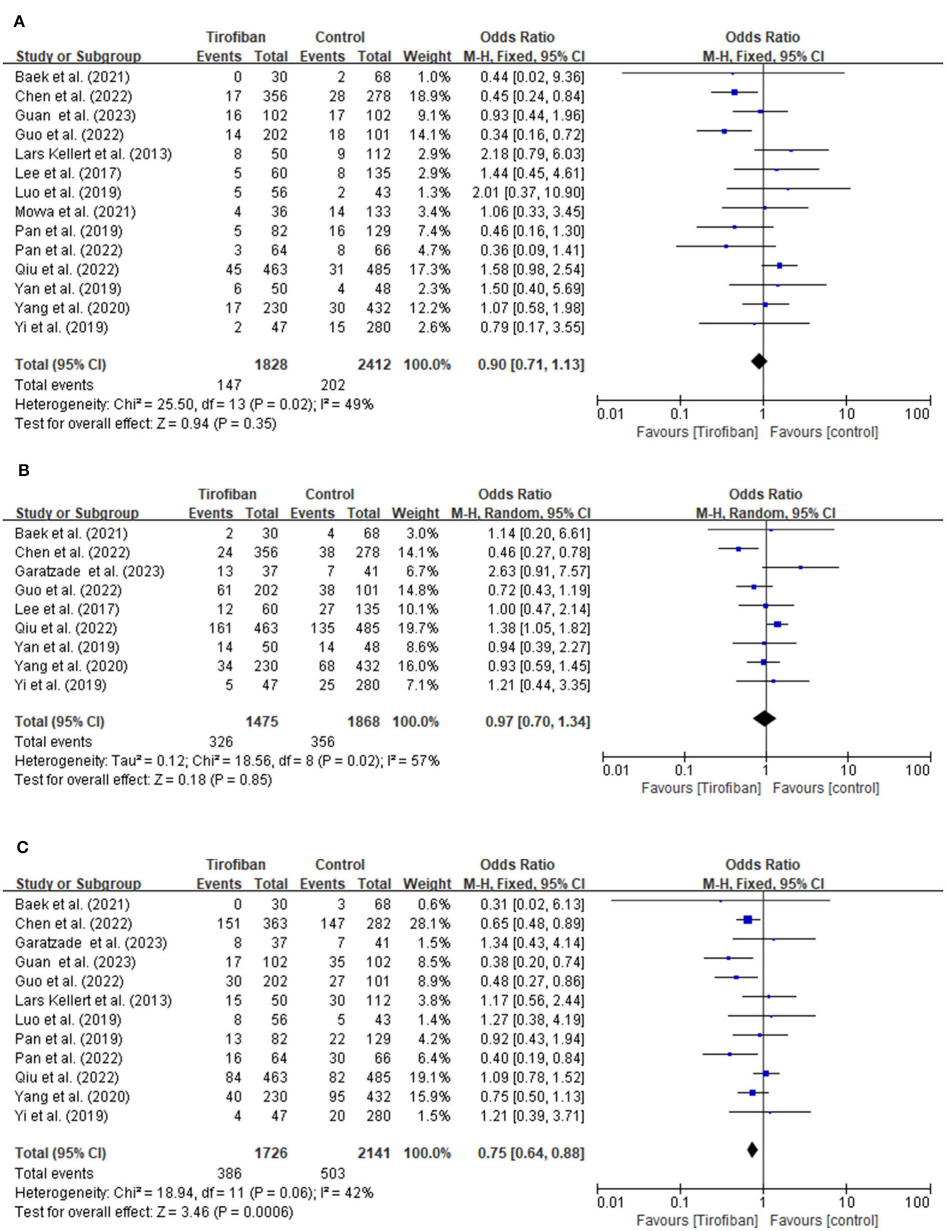
**(A, B)** Forest plot of tirofiban vs. control for safety outcomes meta-analyses of related studies.

Fourteen studies document the occurrences of sICH (Figure 2A). The incidence of sICH is 8.0% (147/1,828) in the tirofiban group compared to 8.4% (202/2,412) in the non-tirofiban group. No significant difference is observed when estimating the relationship between tirofiban and sICH for patients with AIS treated by ET (OR, 0.90; 95% CI, 0.71–1.13; *p* = 0.35). The heterogeneity between the two groups is low (*p* = 0.35; I^2^ = 49%).

#### 3.3.2 Any ICH incidence

A total of nine studies examine the incidence of any ICH (Figure 2B). The tirofiban group comprises 326 out of 1,475 (22.1%) patients, whereas the non-tirofiban group includes 356 out of 1,868 (19.1%) patients. The pooled analysis reveals no significant difference in the incidence of any ICH (OR, 0.97; 95% CI, 0.70–1.34; *p* = 0.85). The heterogeneity between the two groups is high (*p* = 0.02; I^2^ = 57%).

#### 3.3.3 90-day mortality

The 90-day mortality is recorded in 12 studies (Figure 2C). Within 90 days, 386 individuals (16.7%) in the tirofiban group and 503 individuals (17.2%) in the non-tirofiban group died. Compared to the non-tirofiban group, the mortality in the tirofiban group is lower (OR, 0.75; 95% CI, 0.64–0.88; *p* = 0.0006). Significant heterogeneity is not observed (*p* = 0.06; I^2^ = 42%).

### 3.4 Subgroup analysis

**Figure 3:**
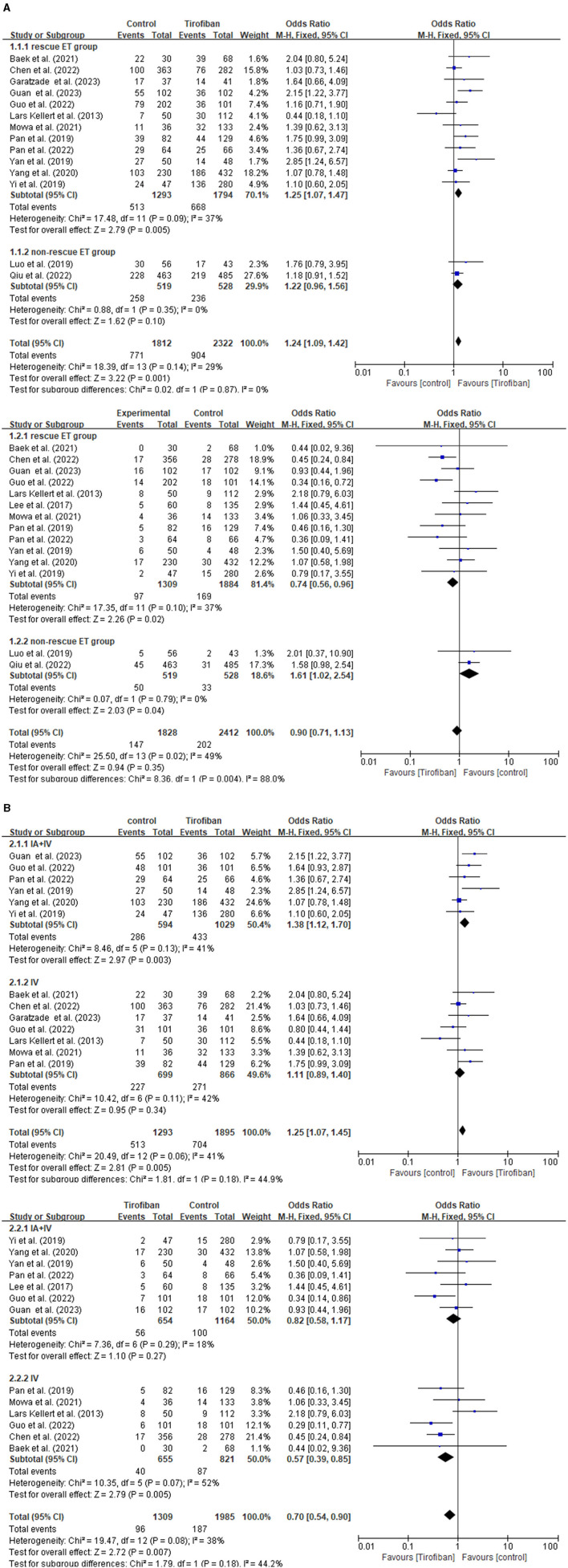
Forest plot of the favorable functional outcome and the symptomatic intracranial hemorrhage in subgroup analysis. **(A)** Rescue ET group vs. non-rescue ET group. **(B)** Combining intra-arterial with intravenous tirofiban vs. intravenous tirofiban alone in rescue ET group.

The subgroup analysis is conducted by categorizing the 15 studies into rescue ET group and non-rescue ET group (Figure 3A). This analysis demonstrates that tirofiban exhibits more favorable prospects in the rescue ET group, as it led to better prognosis (OR, 1.24; 95% CI, 1.09–1.42; p = 0.001) without increasing the risk of sICH (OR, 0.90; 95% CI, 0.71–1.13; *p* = 0.35).

Furthermore, another subgroup analysis is conducted to investigate the effect of tirofiban administration in the rescue ET group (Figure 3B). Two categories of studies are identified, such as intravenous tirofiban alone, and a combination of intra-arterial and intravenous tirofiban. Patients receiving a combination of intra-arterial and intravenous tirofiban experience a significantly improved favorable functional outcome (OR, 1.25; 95% CI, 1.07–1.45; *p* = 0.005) without increasing the risk of sICH (OR, 0.70; 95% CI, 0.54–0.90; *p* = 0.007) in the rescue ET group.

### 3.5 Sensitivity analysis

No significant heterogeneity is observed among the studies regarding favorable functional outcome, excellent functional outcome, 90-day mortality, and sICH. However, the result for any ICH shows heterogeneity. Therefore, the sensitivity analysis is conducted by excluding studies one by one. The results for the above endpoints are largely stable (Supplementary material 6). Moreover, upon excluding the study by Chen et al. (20), the I^2^ values of any ICH decreased from 57% to 19%. Upon visual inspection of the funnel plot, no apparent asymmetry is observed (Supplementary material 7).

## 4 Discussion

The primary objective of the present analysis is to evaluate the efficacy and safety of continuous intravenous tirofiban in patients with AIS who undergo ET. According to the results, receiving continuous intravenous tirofiban is associated with a better prognosis without increasing the risk of sICH for patients with AIS undergoing rescue ET, and the combined use of intra-arterial tirofiban particularly demonstrates significant efficacy.

This pooled analysis reveals that continuous intravenous tirofiban is linked to improved functional outcomes. It is well-established that ET can benefit patients with AIS caused by LVO by restoring blood flow reperfusion. In other words, recanalization rates have been steadily enhanced to over 80% through the utilization of various thrombectomy techniques (27). However, regrettably, less than half of the patients with positive reperfusion in the large vasculature experience good prognosis (2). The release and expansion of the stent and balloon during the procedure lead to plaque compression, fragmentation, and shedding, leading to microemboli formation within the distal microvasculature and resulting in inadequate perfusion (28). Additionally, the mechanical thrombectomy device can exert abrasive force, causing endothelial injury and subsequent reocclusion (29). The aforementioned factors may contribute to the poor functional outcome. In this regard, tirofiban plays a crucial role in improving the prognosis of patients with AIS undergoing ET due to its rapid and potent antiplatelet effects.

The current study highlights the significant importance of continuous intravenous tirofiban in improving the prognosis of patients with AIS undergoing ET. However, Qiu et al. demonstrated that administration of intravenous tirofiban did not have a significant impact on enhancing long-term patient outcomes during ET for patients with AIS, although there was slight tendency toward a more favorable result (4). In other words, as the sample size expands, the study may yield different conclusions. Several meta-analyses have examined the efficacy and safety of continuous intravenous tirofiban in patients with AIS undergoing ET (5, 6). However, there is a lack of comprehensive research specifically focusing on the route of administration of tirofiban. Zhang et al. demonstrated that intravenous administration of tirofiban remains both safe and efficacious in treating patients with AIS who undergo ET (30), which is consistent with the conclusion of this study. Therefore, combining the recent evidence, this study shows continuous intravenous tirofiban during ET is particularly important for enhancing the prognosis of patients with AIS.

The Safety of Tirofiban in Acute Ischemic Stroke (SaTIS) trial confirms that continuous intravenous infusion of tirofiban is safe for patients with AIS (31). In line with the aforementioned outcome, the present meta-analysis reveals that the administration of continuous intravenous tirofiban to patients with AIS undergoing ET does not increase the risk of hemorrhage or the 90-day mortality. However, there is an ongoing debate regarding whether the administration of continuous intravenous tirofiban enhances the risk of hemorrhage in patients with AIS undergoing ET. In a cohort study, Kellert et al. concluded that tirofiban treatment during ET was associated with a higher risk of developing fatal ICH and poor outcomes (23). Several factors may contribute to this contradiction. Kellert et al. collected data from 2006 to 2011 (23), while other studies included are conducted after 2015 when there are significant improvements in technology and devices, such as mechanical thrombectomy, ballooning, and stenting. Furthermore, there have been enhancements in patient screening criteria. Nevertheless, Wu et al. stated that tirofiban exhibits a dose-dependent impact on ICH (32), with a higher incidence of hemorrhage observed at higher dosages of tirofiban. Therefore, in recent years, most studies have opted for low-dose tirofiban. Consistent with these findings, the data demonstrate no correlation between continuous intravenous administration of low-dose tirofiban and the risks of developing sICH, any ICH, or 90-day mortality.

For patients with AIS caused by LVO, ET becomes the preferred treatment option. The standard ET typically involves stent retriever thrombectomy and aspiration thrombectomy. However, it has been observed in clinical practice that a small subset of patients are unable to achieve successful recanalization (Thrombolysis in Cerebral Infarction 2b-3) following the positive standard ET. Therefore, the rescue ET is performed, wherein procedures, such as angioplasty and stent placement, are applied as rescue measures in cases of failed recanalization. Tirofiban would serve as a rescue medication under the following conditions: (1) severe residual stenosis after thrombectomy; (2) treatment with emergency stenting or balloon angioplasty for failed thrombectomy; (3) successful recanalization achieved after at least three attempts with a high potential for endothelial injuries; and (4) severe atherosclerosis with a high risk of reocclusion. The subgroup analysis reveals that tirofiban exhibits more favorable prospects in the rescue ET, demonstrating improved prognosis without increasing the risk of sICH.

This study is the first meta-analysis to demonstrate a significant enhancement in the proportion of individuals achieving functional independence when continuous intravenous tirofiban are used in the rescue ET, especially combined with intra-arterial injection. The present findings provide new evidence supporting the clinical use of combining intra-arterial injection with continuous intravenous tirofiban in patients with AIS undergoing rescue ET. Previous studies have found that there is a risk of reocclusion in patients with AIS undergoing ET, which is a matter of significant concern (3, 33). This reocclusion negatively affects the functional outcome of patients with AIS who undergo ET. In such cases, the significance of rescue ET should not be overlooked. Tirofiban administration via the target artery prevents local thrombosis and arterial reocclusion by directly acting on the injured endothelium and dissolving plaque. Additionally, the concentrated dose of tirofiban delivered to the distal end with the blood flow plays a crucial role in alleviating microthrombus formation and improving microcirculation. Continuous intravenous tirofiban may assist in maintaining sufficiently high blood concentrations to prevent platelet aggregation. Previous cohort studies conducted by Yan et al. and Guo et al. have reported that combining intra-arterial with intravenous tirofiban is associated with improvements in the functional outcomes of patients undergoing rescue ET, which aligns with the findings of the present analysis (14, 17). Overall, this study provides valuable insights into the combination of intra-arterial injection and intravenous tirofiban administration in patients treated by rescue ET, demonstrating its efficacy and safety while highlighting its potential to improve functional outcomes without increasing the risk of sICH. Therefore, further research should focus on this combination therapy in patients with AIS undergoing rescue ET.

This analysis has several limitations as well. First, the majority of the included studies are cohort studies, with only one being RCT. This introduces the possibility of selection bias in the individual patient pool. Nevertheless, the efficacy and safety of continuous intravenous tirofiban remain significant even after conducting the sensitivity analysis and heterogeneity testing. Second, there are differences in baseline characteristics among the included studies, including ET strategies, occlusion locations, and intravenous thrombolysis. Regrettably, the unavailability of individual stratified data limited the ability to adjust for these differences, which may have potentially influenced the conclusions of the present work. Therefore, it is crucial to conduct extensive RCTs with a large number of participants to establish the efficacy and safety of the combination of intra-arterial and intravenous tirofiban in patients with AIS treated by rescue ET.

In conclusion, continuous intravenous tirofiban for patients with AIS treated by rescue ET is effective and safe, particularly when combined with intra-arterial tirofiban, in improving the 3-month favorable functional outcome without increasing the risk of sICH.

## Data availability statement

The original contributions presented in the study are included in the article/Supplementary material, further inquiries can be directed to the corresponding author/s.

## Author contributions

MW: Conceptualization, Data curation, Formal analysis, Methodology, Software, Writing – original draft, Writing – review & editing. JL: Conceptualization, Methodology, Project administration, Supervision, Writing – review & editing. LZ: Methodology, Validation, Visualization, Writing – review & editing. NL: Conceptualization, Data curation, Supervision, Writing – review & editing. XL: Funding acquisition, Investigation, Validation, Writing – review & editing. PW: Conceptualization, Funding acquisition, Investigation, Resources, Software, Supervision, Validation, Writing – original draft, Writing – review & editing.

## References

[1] Group WGftB Zi W Qiu Z Wu D Li F Liu H . Assessment of endovascular treatment for acute basilar artery occlusion via a nationwide prospective registry. JAMA Neurol. (2020) 77:561–73. 10.1001/jamaneurol.2020.0156 32080711 PMC7042866

[2] van Horn N Kniep H Leischner H McDonough R Deb-Chatterji M Broocks G . Predictors of poor clinical outcome despite complete reperfusion in acute ischemic stroke patients. J Neurointervent Surg. (2021) 13:14. 10.1136/neurintsurg-2020-015889 32414889

[3] Marto JP Strambo D Hajdu SD Eskandari A Nannoni S Sirimarco G . Twenty-four-hour reocclusion after successful mechanical thrombectomy: associated factors and long-term prognosis. Stroke. (2019) 50:2960–3. 10.1161/STROKEAHA.119.026228 31535931

[4] Qiu Z Li F Sang H Luo W Liu S Liu W Guo Z . Effect of intravenous tirofiban vs placebo before endovascular thrombectomy on functional outcomes in large vessel occlusion stroke the RESCUE BT randomized clinical trial. JAMA. (2022) 328:543–53. 35943471 10.1001/jama.2022.12584 PMC9364124

[5] Fu Z Xu C Liu X Wang Z Gao L. Safety and efficacy of tirofiban in acute ischemic stroke patients receiving endovascular treatment: a meta-analysis. Cerebrovasc Dis. (2020) 49:442–50. 10.1159/000509054 32731250

[6] Sun Y Guo ZN Yan X Wang M Zhang P Qin H . Safety and efficacy of tirofiban combined with endovascular therapy compared with endovascular therapy alone in acute ischemic stroke: a meta-analysis. Neuroradiology. (2021) 63:17–25. 10.1007/s00234-020-02530-9 32844236

[7] Page MJ McKenzie JE Bossuyt PM Boutron I Hoffmann TC Mulrow CD . The PRISMA 2020 statement: an updated guideline for reporting systematic reviews. Int J Surg. (2021) 88:105906. 10.1016/j.ijsu.2021.105906 33789826

[8] Hacke W Kaste M Bluhmki E Brozman M Dávalos A Guidetti D . Thrombolysis with alteplase 3 to 4.5 hours after acute ischemic stroke. N Engl J Med. (2008) 359:1317–29. 10.1056/NEJMoa0804656 18815396

[9] von Kummer R Broderick JP Campbell BCV Demchuk A Goyal M Hill MD . The Heidelberg bleeding classification: classification of bleeding events after ischemic stroke and reperfusion therapy. Stroke. (2015) 46:2981–6. 10.1161/STROKEAHA.115.010049 26330447

[10] Stang A. Critical evaluation of the Newcastle-Ottawa scale for the assessment of the quality of nonrandomized studies in meta-analyses. Eur J Epidemiol. (2010) 25:603–5. 10.1007/s10654-010-9491-z 20652370

[11] Adams HP Jr Bendixen BH Kappelle LJ Biller J Love BB Gordon DL . Classification of subtype of acute ischemic stroke. Definitions for use in a multicenter clinical trial. TOAST. Trial of Org 10172 in Acute Stroke Treatment. Stroke. (1993) 24:35–41. 10.1161/01.STR.24.1.35 7678184

[12] Sterne JAC Savovic J Page MJ Elbers RG Blencowe NS Boutron I . RoB 2: a revised tool for assessing risk of bias in randomised trials. BMJ. (2019) 366:l4898. 10.1136/bmj.l4898 31462531

[13] Baek BH Yoon W Lee YY Kim SK Kim JT Park MS. Intravenous tirofiban infusion after angioplasty and stenting in intracranial atherosclerotic stenosis-related stroke. Stroke. (2021) 52:1601–8. 10.1161/STROKEAHA.120.033551 33793319

[14] Guo W Xu J Ma L Ma J Li S Ren C . Safety and efficacy of different tirofiban administration routes on acute ischemic stroke patients with successful recanalization: a propensity score matching analysis. CNS Neurosci Ther. (2022) 28:1993–2000. 10.1111/cns.13936 35962605 PMC9627363

[15] Lee J-I Gliem M Gerdes G Turowski B Kaschner M Kraus B . Safety of bridging antiplatelet therapy with the gpIIb-IIIa inhibitor tirofiban after emergency stenting in stroke. PLoS ONE. (2017) 12:190218. 10.1371/journal.pone.0190218 29281734 PMC5745002

[16] Movva H Rabah R Tekle W Preston L Kotto H Hassan AE. There is no difference in safety and efficacy mechanical thrombectomy alone or mechanical thrombectomy with tirofiban for patients undergoing treatment of large vessel occlusion and underlying intracranial atherosclerosis. Interdiscipl Neurosurg. (2022) 27:101383. 10.1016/j.inat.2021.101383

[17] Yan Z Shi Z Wang Y Zhang C Cao J Ding C . Efficacy and Safety of Low-Dose Tirofiban for Acute Intracranial Atherosclerotic Stenosis Related Occlusion with Residual Stenosis after Endovascular Treatment. J Stroke Cerebrovas Dis. (2020) 29:104619. 10.1016/j.jstrokecerebrovasdis.2019.104619 31982305

[18] Yi HJ Sung JH Lee DH. Safety and efficacy of intra-arterial tirofiban injection during mechanical thrombectomy for large artery occlusion. Curr Neurovasc Res. (2019) 16:416–24. 10.2174/1567202616666191023154956 31702492

[19] Luo Y Yang Y Xie Y Yuan Z Li X Li J. Therapeutic effect of pre-operative tirofiban on patients with acute ischemic stroke with mechanical thrombectomy within 6-24 hours. Intervent Neuroradiol. (2019) 25:705–9. 10.1177/1591019919851167 31112428 PMC6838844

[20] Chen Q Meng R Wu D Hu J Tao Z Xie D . Association of intravenous tirofiban with functional outcomes in acute ischemic stroke patients with acute basilar artery occlusion receiving endovascular thrombectomy. Cerebrovasc Dis. (2023) 52:451–9. 10.1159/000527483 36481613 PMC10568592

[21] Garayzade R Berlis A Schiele S Schneider H Ertl M Müller G . Comparison of safety and efficacy after emergency stenting in patients exhibiting intracranial atherosclerotic stenosis associated with large-vessel occlusion with and without intravenous infusion of tirofiban. Cardiovasc Intervent Radiol. (2023) 46:377–84. 10.1007/s00270-023-03372-7 36797426 PMC10014670

[22] Guan Q Yun W Li X Ni H Lv W Xie Z . Association of tirofiban with improvement of functional outcomes of direct thrombectomy for acute anterior circulation occlusion: a retrospective, nonrandomized, multicenter, real-world study. Neurosurg Focus. (2023) 55:E21. 10.3171/2023.7.FOCUS23150 37778035

[23] Kellert L Hametner C Rohde S Bendszus M Hacke W Ringleb P . Endovascular stroke therapy tirofiban is associated with risk of fatal intracerebral hemorrhage and poor outcome. Stroke. (2013) 44:1453. 10.1161/STROKEAHA.111.000502 23463755

[24] Pan X Zheng D Zheng Y Chan PWL Lin Y Zou J . Safety and efficacy of tirofiban combined with endovascular treatment in acute ischaemic stroke. Eur J Neurol. (2019) 26:1105–10. 10.1111/ene.13946 30793464

[25] Yang M Huo X Gao F Wang A Ma N Shi H . Low-dose rescue tirofiban in mechanical thrombectomy for acute cerebral large-artery occlusion. Eur J Neurol. (2020) 27:1056–61. 10.1111/ene.14170 32048389

[26] Pan X Xu M Fei Y Lin S Lin Y Zou J . Influence of tirofiban on stroke outcome after mechanical thrombectomy in acute vertebrobasilar artery occlusion. BMC Neurol. (2022) 22:460. 10.1186/s12883-022-02996-5 36494796 PMC9733212

[27] Nogueira RG Jadhav AP Haussen DC Bonafe A Budzik RF Bhuva P . Thrombectomy 6 to 24 Hours after Stroke with a Mismatch between Deficit and Infarct. N Engl J Med. (2018) 378:11–21. 10.1056/NEJMoa1706442 29129157

[28] Wu J Lu AD Zhang LP Zuo YX Jia YP. Study of clinical outcome and prognosis in pediatric core binding factor-acute myeloid leukemia. Zhonghua Xue Ye Xue Za Zhi. (2019) 40:52–7. 10.3760/cma.j.issn.0253-2727.2019.01.010 30704229 PMC7351698

[29] Qureshi AI Siddiqui AM Kim SH Hanel RA Xavier AR Kirmani JF . Reocclusion of recanalized arteries during intra-arterial thrombolysis for acute ischemic stroke. AJNR Am J Neuroradiol. (2004) 25:322–8. 14970040 PMC7974601

[30] Zhang A Wu N Liu X Jiang T. Continuous intravenous tirofiban can improve the 90-day functional outcome and decrease 90-day mortality without increasing bleeding risk in acute ischemic stroke patients treated by endovascular therapy: a meta-analysis. J Clin Neurosci. (2022) 99:109–16. 10.1016/j.jocn.2022.03.008 35278931

[31] Siebler M Hennerici MG Schneider D von Reutern GM Seitz RJ Röther J . Safety of tirofiban in acute ischemic stroke: the SaTIS trial. Stroke. (2011) 42:2388–92. 10.1161/STROKEAHA.110.599662 21852609

[32] Wu Y Yin C Yang J Jiang L Parsons MW Lin L. Endovascular thrombectomy. Stroke. (2018) 49:2783–5. 10.1161/STROKEAHA.118.022919 30355186

[33] Janjua N Alkawi A Suri MFK Qureshi AI. Impact of arterial reocclusion and distal fragmentation during thrombolysis among patients with acute ischemic stroke. AJNR Am J Neuroradiol. (2008) 29:253–8. 10.3174/ajnr.A0825 18024576 PMC8119013

