# Supplementary material for: The efficacy and safety of continuous intravenous tirofiban for acute ischemic stroke patients treated by endovascular therapy: a meta-analysis
Source: Front Neurol. 2024 Apr 3;15:1286079. doi: 10.3389/fneur.2024.1286079 (PMC11021731; doi:10.3389/fneur.2024.1286079)
Supplement: Supplementary file 2 [file Table_2.docx]

**Supplementary Material 2.** Flowchart of the study selection process.


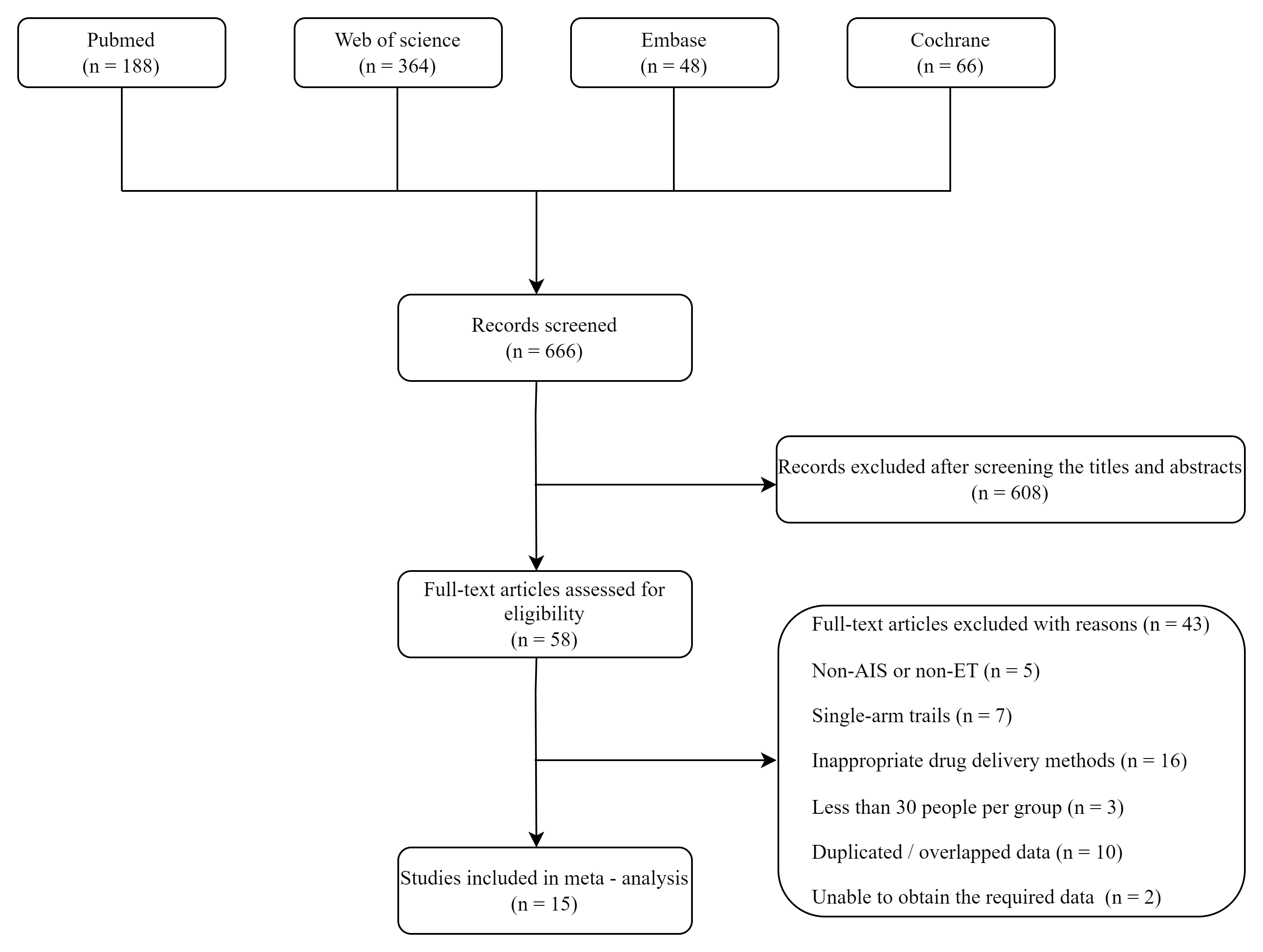
**Abbreviations:** AIS, acute ischemic stroke; ET, endovascular therapy.
