# Supplementary material for: The efficacy and safety of continuous intravenous tirofiban for acute ischemic stroke patients treated by endovascular therapy: a meta-analysis
Source: Front Neurol. 2024 Apr 3;15:1286079. doi: 10.3389/fneur.2024.1286079 (PMC11021731; doi:10.3389/fneur.2024.1286079)
Supplement: Supplementary file 3 [file Table_3.docx]

**Supplementary Material 3** Baseline characteristics of the patients with acute ischemic stroke.

| author (year) | country | study design | sample size | | sex (male) | | age, mean | | NHISS at arrival, mean | | IVT | | OTR, IQR | | OTP, IQR | | administration route | | | cause of stroke | | | | | | | efficacy outcomes | | | | safe outcomes | | | | | |
| --- | --- | --- | --- | --- | --- | --- | --- | --- | --- | --- | --- | --- | --- | --- | --- | --- | --- | --- | --- | --- | --- | --- | --- | --- | --- | --- | --- | --- | --- | --- | --- | --- | --- | --- | --- | --- |
|  |  |  | T | N | T | N | T | N | T | N | T | N | T | N | T | N | IA | IV | LAA | | | CE | | Other | | mRS 0-1 | | | mRS 0-2 | | any ICH | | sICH | | 90-day mortality | |
|  |  |  |  |  |  |  |  |  |  |  |  |  |  |  |  |  |  |  | T | | N | T | N | T | N | T | | N | T | N | T | N | T | N | T | N |
| Baek et al. (2021) | Korea | R | 30 | 68 | 16 | 42 | 61.9 | 64.8 | 10.3 | 10.7 | 9 | 26 | NA | NA | NA | NA | NA | continuous infusion of 0.1 µg/kg/min for 12 h | NA | | NA | NA | NA | NA | NA | 12 | | 23 | 22 | 39 | 2 | 4 | 0 | 2 | 0 | 3 |
| Chen et al. (2022) | China | R | 363 | 282 | 282 | 200 | 63.4 | 65.7 | 25.6 | 25.6 | 62 | 57 | 451 (323-644) | 430 (334-621) | 250 (122-410) | 241 (141-373) | NA | 0.4 µg/kg/min for 30 min and then continuous infusion of 0.1µg/kg/min for 24 h | 275 | | 142 | 59 | 113 | 29 | 27 | 69 | | 64 | 100 | 76 | 24 | 38 | 17 | 28 | 151 | 147 |
| Garayzade et al. (2023) | Germany | R | 37 | 41 | 27 | 21 | 69.6 | 71.8 | NA | NA | 11 | 21 | NA | NA | NA | NA | NA | a dose of 25 mcg/kg within 3 min and then continuous infusion of 0.15 mcg/kg/min for 24 h | NA | | NA | NA | NA | NA | NA | NA | | NA | 17 | 14 | 13 | 7 | NA | NA | 8 | 7 |
| Guan et al. (2023) | China | R | 102 | 102 | 73 | 56 | 67.4 | 72.8 | NA | NA | NA | NA | 355 (253-421) | 300 (235-360) | 214 (150-300) | 210 (150-271) | a bolus dose of 0.25-1 mg | continuous infusion of 0.1 µg/kg/min for 12-24 h | 32 | | 24 | 58 | 65 | 12 | 13 | 38 | | 30 | 55 | 36 | NA | NA | 16 | 17 | 17 | 25 |
| Guo et al. (2022) | China | R | 202 | 101 | 157 | 81 | NA | NA | NA | NA | 66 | 32 | NA | NA | NA | NA | a bolus dose of 0.25-0.5 mg IA or IV | continuous infusion of 0.2-0.4 mg/h for 12-24 h | 197 | | 238 | 212 | 194 | 16 | 14 | 52 | | 25 | 79 | 36 | 61 | 38 | 14 | 18 | 30 | 27 |
| Lars Kellert et al. (2013) | Germany | P | 50 | 112 | NA | NA | 64.5 | 67.3 | 22.2 | 19.4 | 35 | 79 | 254 (157-353) | 239 (164-386) | 140 (93-270) | 150 (92-280) | NA | adapted for weight and creatinine clearance for at least 12 h | NA | | NA | NA | NA | NA | NA | 4 | | 13 | 7 | 30 | NA | NA | 8 | 9 | 15 | 30 |
| Lee et al. (2017) | Germany | R | 60 | 135 | 38 | 55 | 69.6 | 75.4 | 11 | 14.6 | 48 | 116 | 263 (217-332) | 264 (207-356) | 175 (139-238) | 183 (143-294) | a bolus dose of 1.250 mg | continuous infusion of 0.1µg/kg/min for 12 h | NA | | NA | NA | NA | NA | NA | NA | | NA | NA | NA | 12 | 27 | 5 | 8 | NA | NA |
| Luo et al. (2019) | China | R | 56 | 43 | 36 | 24 | 64 | 68 | 16.4 | 15.7 | NA | NA | NA | NA | NA | NA | NA | continuous infusion 50 mg/kg at 50 mg/min | NA | | NA | NA | NA | NA | NA | NA | | NA | 30 | 17 | NA | NA | 5 | 2 | 8 | 5 |
| Movva et al. (2021) | United States | R | 36 | 133 | 26 | 73 | 69.3 | 67.9 | 13.1 | 17.9 | 5 | 51 | NA | NA | NA | NA | a bolus dose of 12mcg/kg over 30 min | continuous infusion of 0.1 mcg/kg/min for 6–12 h | NA | | NA | NA | NA | NA | NA | NA | | NA | 11 | 32 | NA | NA | 4 | 14 | NA | NA |
| Pan et al. (2019) | China | P | 82 | 129 | 52 | 79 | 70.8 | 72.6 | 14.6 | 14.9 | 28 | 94 | NA | NA | 215 (155-345) | 180 (120-245) | NA | continuous infusion of 0.15 μg/kg/min for 16–24 h | 65 | | 10 | 35 | 63 | 2 | 8 | NA | | NA | 39 | 44 | NA | NA | 5 | 16 | 13 | 22 |
| Pan et al. (2022) | China | P | 64 | 66 | 50 | 46 | 65.8 | 67.1 | 18.8 | 20.3 | 16 | 26 | NA | NA | 314 (195-436) | 240 (155-405) | a bolus dose of 0.25-1 mg | continuous infusion of 0.1-0.15 µg/kg/min for 16-24 h | 47 | | 35 | 11 | 25 | 6 | 6 | NA | | NA | 29 | 25 | NA | NA | 3 | 8 | 16 | 30 |
| Qiu et al. (2022) | China | RCT | 463 | 485 | 263 | 294 | 72.6 | 66.3 | 15.6 | 16 | 0 | 948 | 490 (340-717) | 481 (314-732) | 400 (272-627) | 398 (246-618) | NA | a bolus dose of 10 μg/kg and then continuous infusion of 0.15 μg/kg/min for up to 24 h | 197 | | 238 | 212 | 194 | 16 | 14 | 168 | | 157 | 228 | 219 | 161 | 135 | 45 | 31 | 84 | 82 |
| Yan et al. (2019) | China | R | 50 | 48 | 29 | 28 | 68.1 | 70.5 | 19 | 21.4 | 43 | 34 | 257 (201-301) | 299 (220-323) | NA | NA | a bolus dose ranged from 0.4-0.5 mg at 0.05 mg/min | continuous infusion of 0.4-0.5 mg/h for 24 h | NA | | NA | NA | NA | NA | NA | 12 | | 8 | 27 | 14 | 14 | 14 | 6 | 4 | NA | NA |
| Yang et al. (2020) | China | P | 230 | 432 | 161 | 271 | 62.9 | 64.7 | 17.1 | 16.7 | 55 | 152 | 395 (300-490) | 360 (290-450) | 300 (200-400) | 262 (197-355) | a bolus dose of 0.25-1 mg | continuous infusion of 0.1 µg/kg/min for 24 h | 166 | | 246 | 33 | 106 | 31 | 80 | NA | | NA | 103 | 186 | 34 | 68 | 17 | 30 | 40 | 95 |
| Yi et al. (2019) | Korea | R | 47 | 280 | 28 | 157 | 73.4 | 69.1 | 10.5 | 9.6 | 22 | 111 | NA | NA | NA | NA | a bolus dose of 0.25 mg at 0.05 mg/min | continuous infusion of 0.05 µg/kg/min for 24 h | NA | | NA | NA | NA | NA | NA | NA | | NA | 24 | 136 | 5 | 25 | 2 | 15 | 4 | 20 |

**Abbreviations**: T: Tirofiban group; N: non-Tirofiban group; NIHSS: National Institutes of Health Stroke Scale; IVT: Intravenous thrombolysis; OTR: onset to recanalization; OTP: onset to puncture; P: Prospective cohort; R: Retrospective cohort; RCT: Randomized controlled trial; IA: Intra-arterial; IV: Intravenous; IQR: interquartile range; NA: not applicable; LAA: Large artery atherosclerosis; CE: Cardioembolism; AC: anterior circulation; PC: posterior Circulation; ICH: intracranial hemorrhage; sICH: symptomatic intracranial hemorrhage; mRS: modified Rankin scale.
