# Supplementary material for: The efficacy and safety of continuous intravenous tirofiban for acute ischemic stroke patients treated by endovascular therapy: a meta-analysis
Source: Front Neurol. 2024 Apr 3;15:1286079. doi: 10.3389/fneur.2024.1286079 (PMC11021731; doi:10.3389/fneur.2024.1286079)

**Supplementary Material 5.** Evaluation of study risk of bias according to the Cochrane Risk of Bias Tool scale.


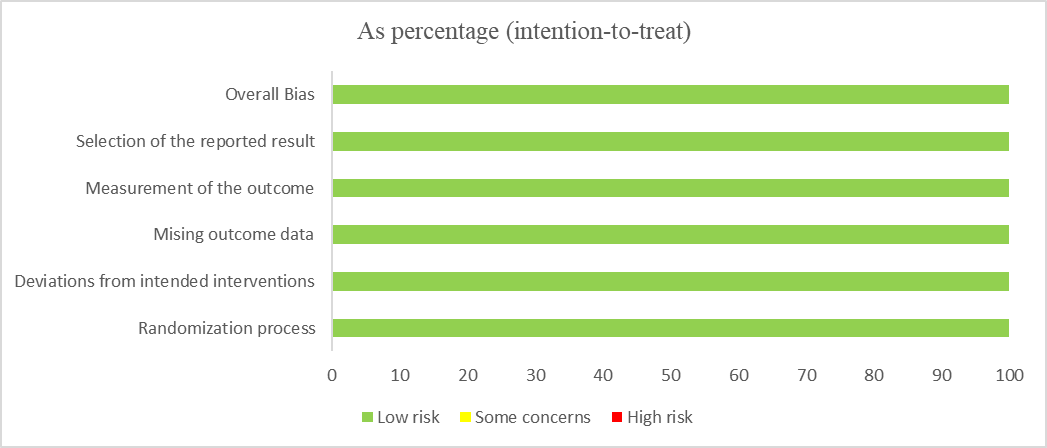

Supplement: Supplementary file 5 [file Table_5.docx]
