# Supplementary material for: The efficacy and safety of continuous intravenous tirofiban for acute ischemic stroke patients treated by endovascular therapy: a meta-analysis
Source: Front Neurol. 2024 Apr 3;15:1286079. doi: 10.3389/fneur.2024.1286079 (PMC11021731; doi:10.3389/fneur.2024.1286079)
Supplement: Supplementary file 6 [file Table_6.docx]

**Supplementary Material 6.** The results of a sensitivity analysis for the efficacy and safety outcomes in acute ischemic stroke patients who underwent endovascular therapy.

(A)
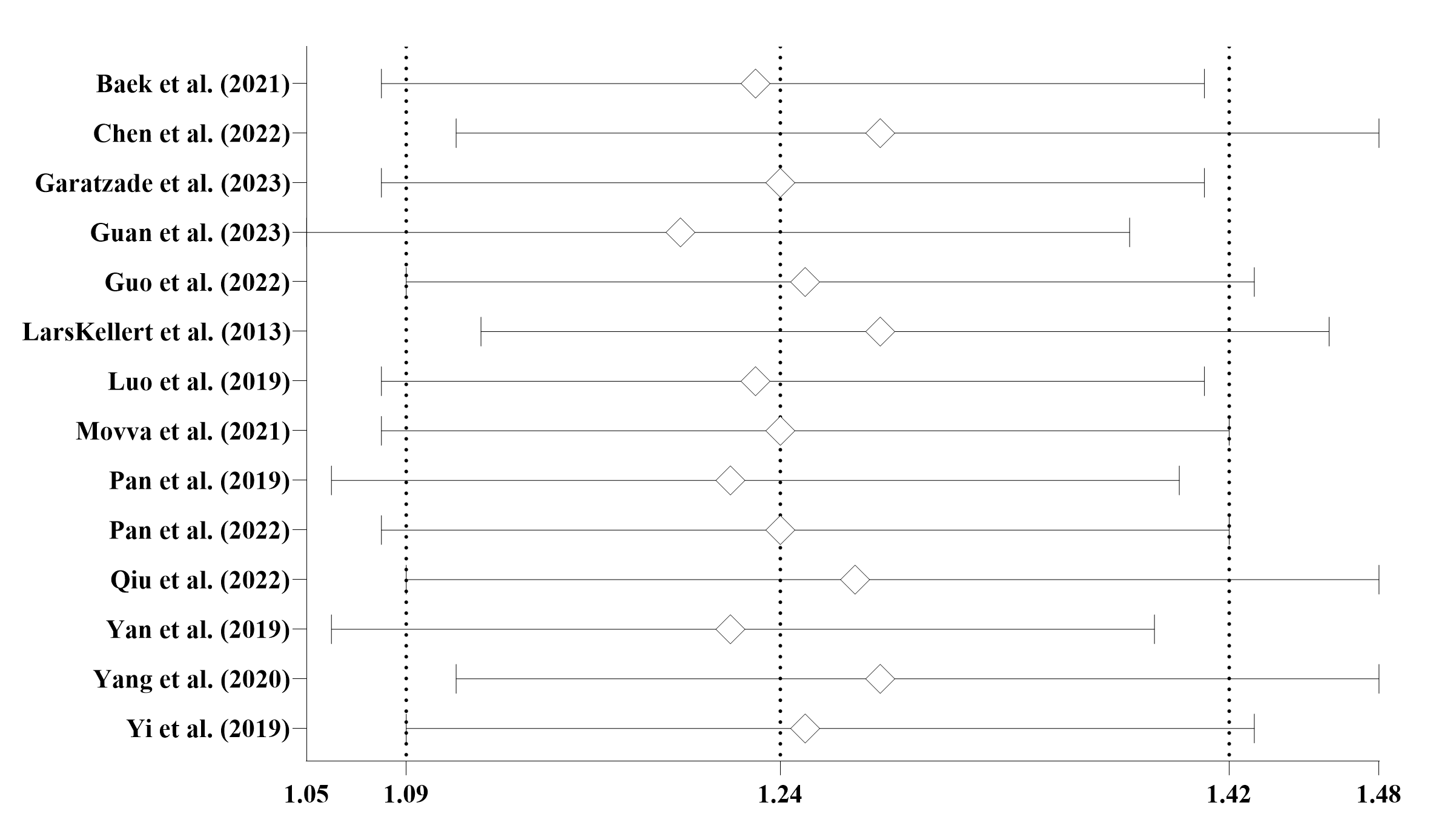


(B)
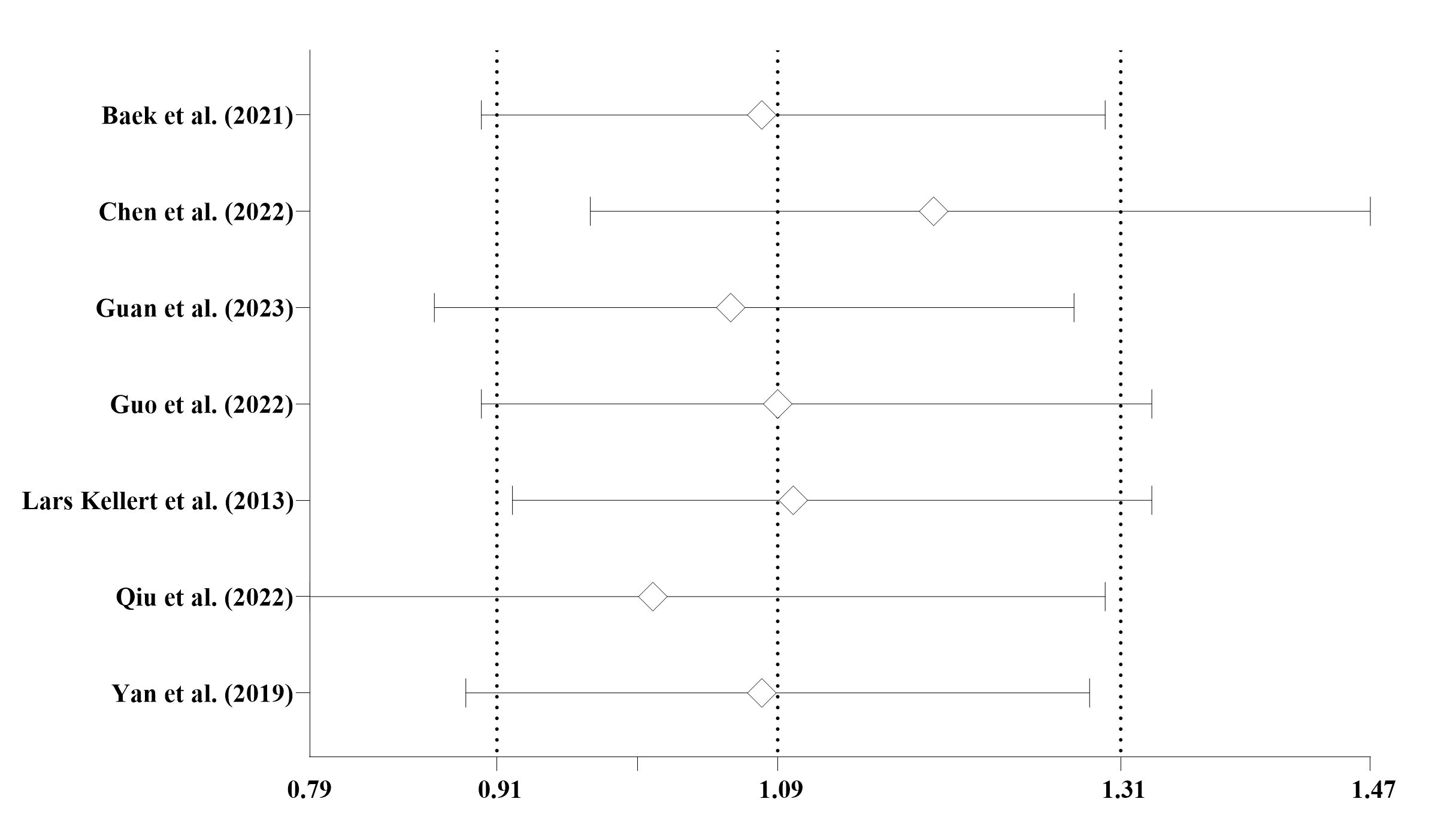


(C)
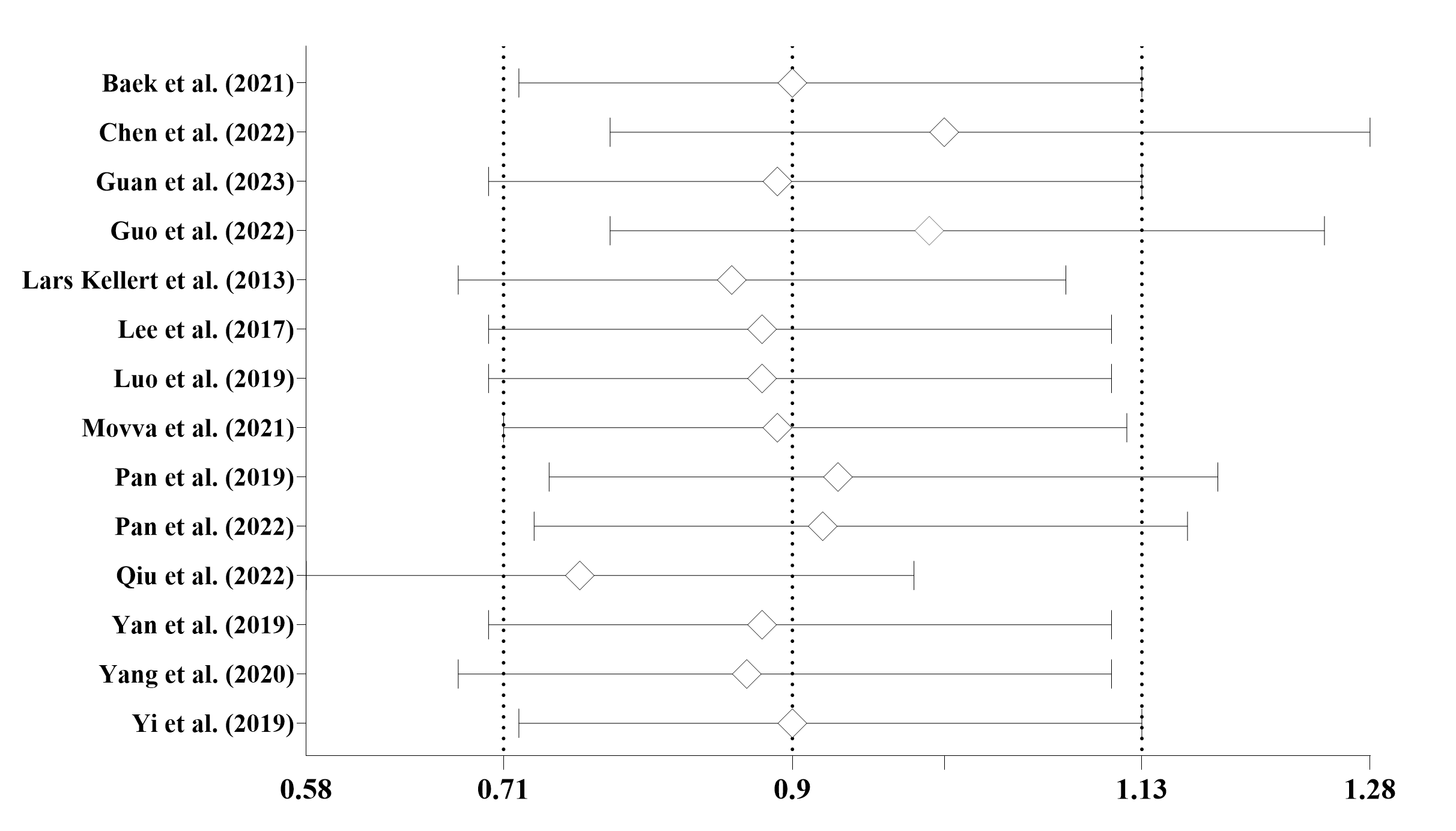


(D)
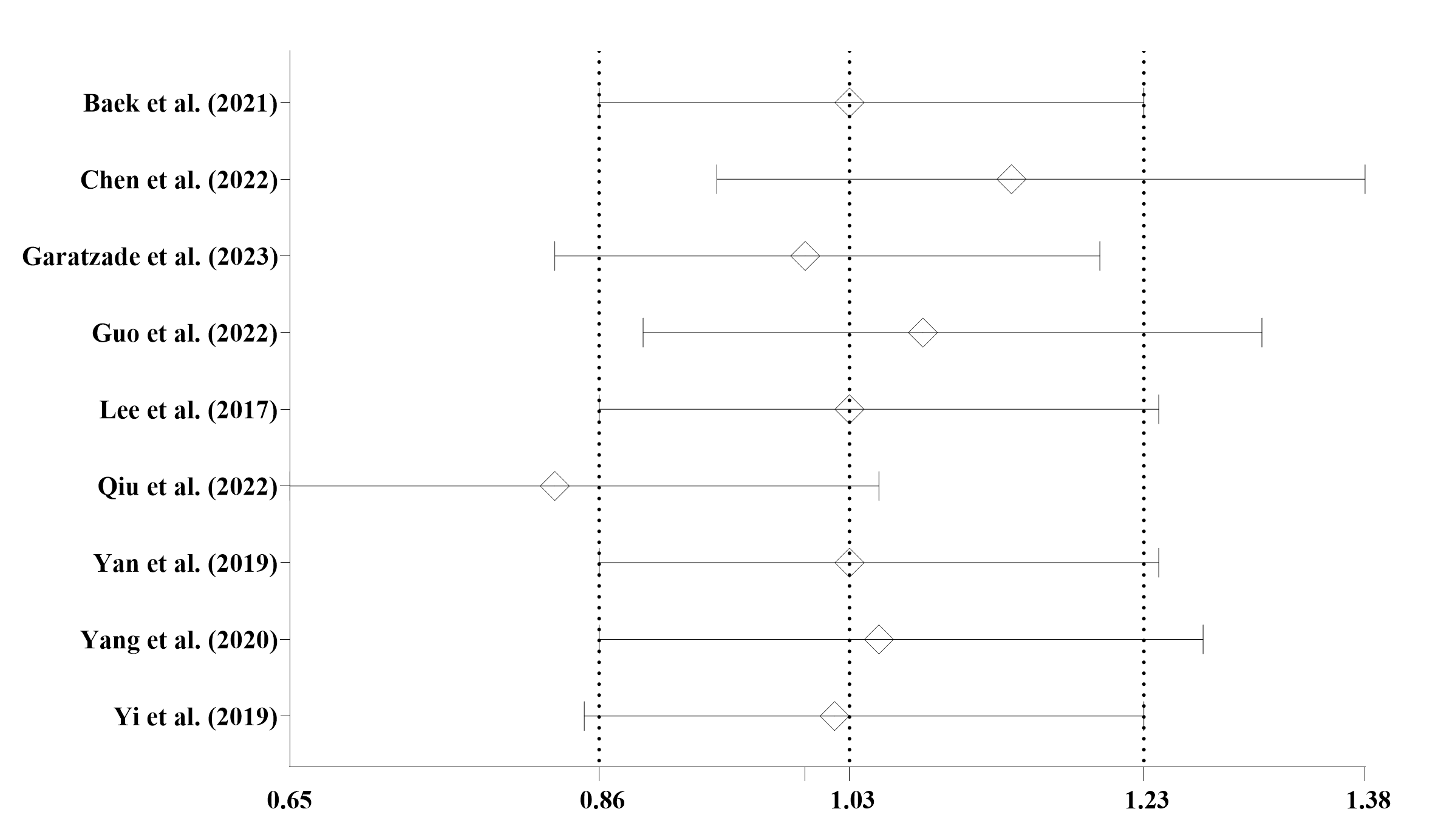


(E)**
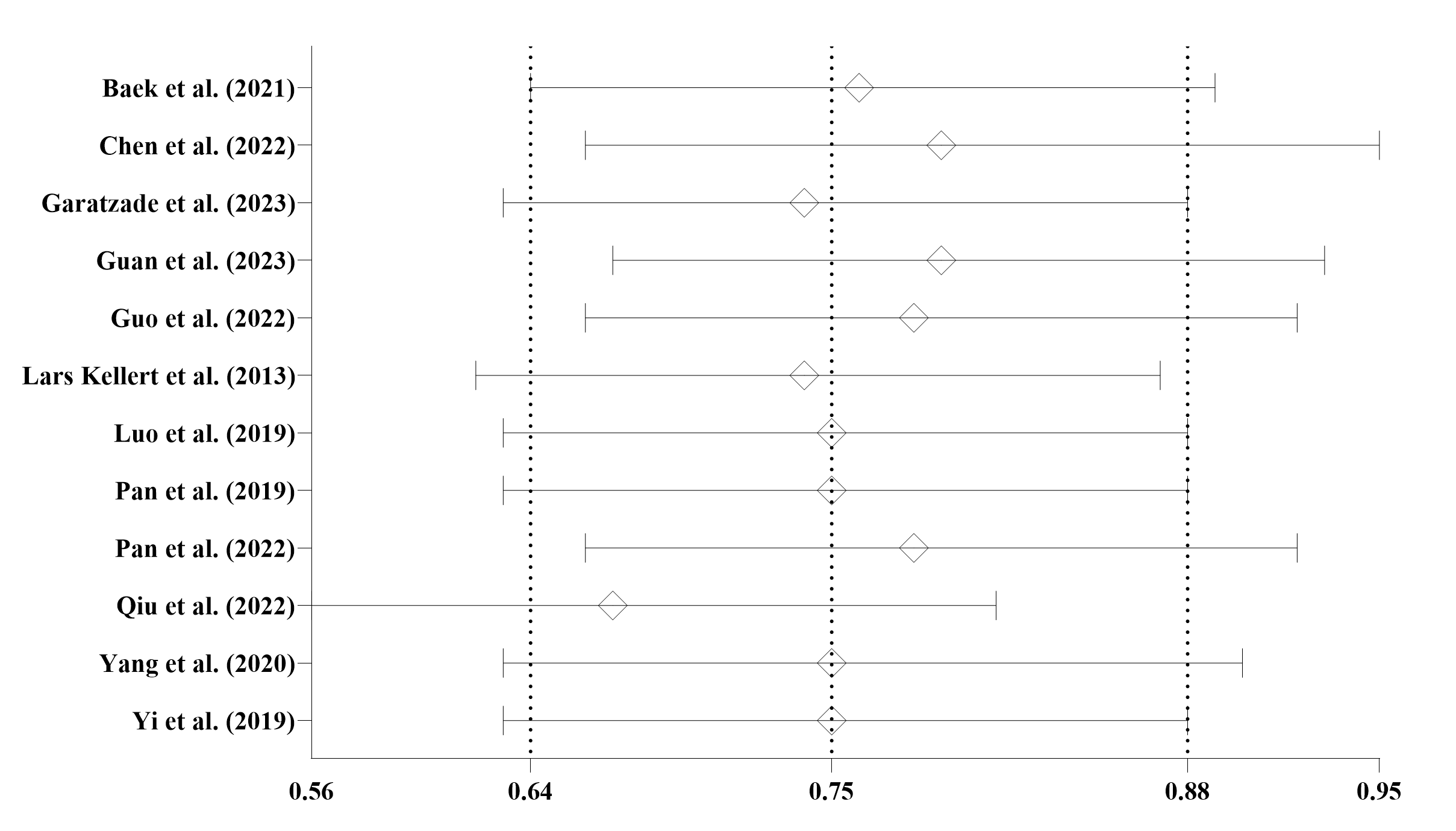
**

(A) The favorable functional outcome. (B) The excellent functional outcome. (C) The symptomatic intracranial hemorrhage. (D) The any intracranial hemorrhage. (E) The 90-day mortality.
