# Supplementary material for: The efficacy and safety of continuous intravenous tirofiban for acute ischemic stroke patients treated by endovascular therapy: a meta-analysis
Source: Front Neurol. 2024 Apr 3;15:1286079. doi: 10.3389/fneur.2024.1286079 (PMC11021731; doi:10.3389/fneur.2024.1286079)
Supplement: Supplementary file 7 [file Table_7.docx]

**Supplementary Material 7.** A funnel plot for assessing publication bias in regards to studies reporting the efficacy and safety outcomes in acute ischemic stroke patients who underwent endovascular therapy.

(A)
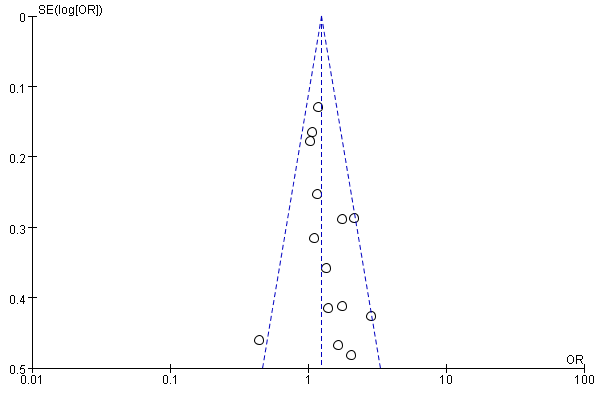
(B)
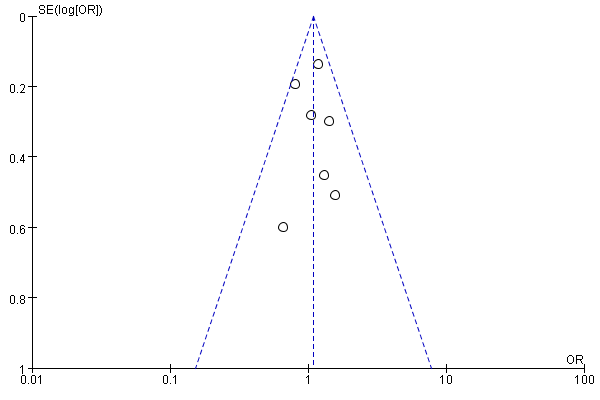


(C)
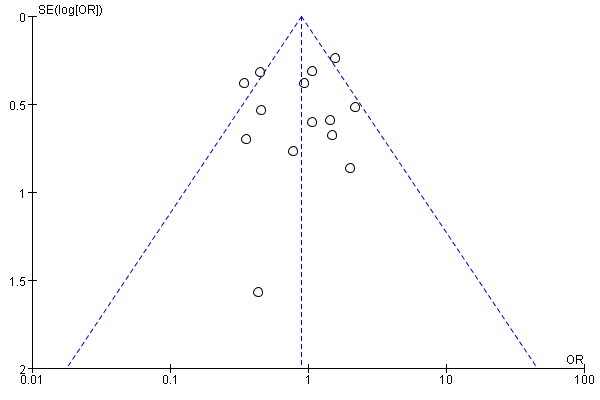


(D)
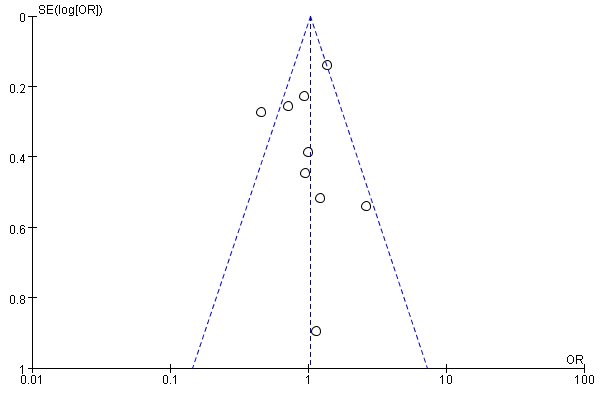


(E)
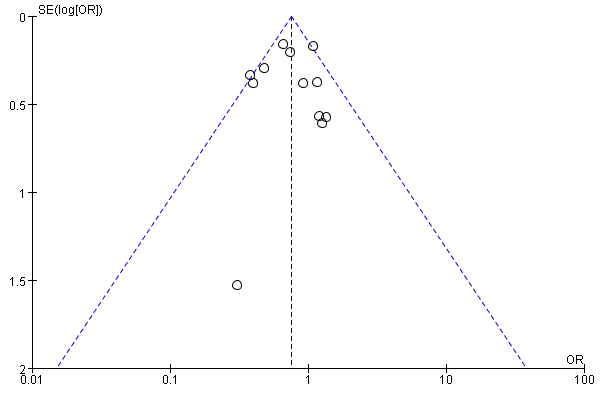
(A) The favorable functional outcome. (B) The excellent functional outcome. (C) The symptomatic intracranial hemorrhage. (D) The any intracranial hemorrhage. (E) The 90-day mortality.
